# A Critical Review of Immunomodulation in the Management of Inoperable Stage III NSCLC

**DOI:** 10.3390/cancers17111829

**Published:** 2025-05-30

**Authors:** Kimberly Burcher, Pooja Karukonda, Christopher Kelsey, Trey Mullikin, Scott J. Antonia, Eziafa I. Oduah

**Affiliations:** Duke Cancer Institute, Durham, NC 27710, USA; kimberly.burcher@duke.edu (K.B.); pooja.karukonda@duke.edu (P.K.); christopher.kelsey@duke.edu (C.K.); trey.mullikin@duke.edu (T.M.); scott.antonia@duke.edu (S.J.A.)

**Keywords:** non-small cell lung cancer, immunomodulation, immunotherapy

## Abstract

The current standard of care for inoperable stage III non-small cell lung cancer (NSCLC) is concurrent chemotherapy and radiation therapy with consolidation durvalumab. Despite this approach, about 50% of patients will experience disease recurrence, with about half of recurrence events occurring at distant metastatic sites. In this review, the authors performed a structured analysis of the available clinical trial data and literature related to the treatment of this disease. The authors discuss the detriments and merits of several of these trials and explore clinical and preclinical data that contribute to the growing body of literature supporting a future with new approaches, including new techniques in radiation therapy, sequencing, and agents. Upcoming trials may illuminate a path towards better outcomes for patients in this setting.

## Simple Summary

The advent of immunotherapy and data published in the landmark PACIFIC trial have revolutionized the treatment of inoperable stage III non-small cell lung cancer (NSCLC). After concurrent chemotherapy and radiation therapy with durvalumab consolidation, however, about 50% of patients will develop disease recurrence, highlighting an ongoing need to improve the outcomes for these patients. This review discusses the history of trials in this space, in addition to strategies to improve outcomes for patients with inoperable stage III NSCLC who are treated according to the current standard of care. The review focuses on strategies related to radiation techniques, sequencing of therapies, and novel agents intended to potentiate the response to immunotherapy. The aim of this review is to guide future basic science research and clinical trials to improve outcomes in this setting.

## 1. Introduction

The past decade has been marked by significant advances in immunotherapy that have transformed the therapeutic landscape of non-small cell lung cancer (NSCLC). Unfortunately, despite this, the overall prognosis of patients with NSCLC is poor regardless of stage. Surgically unresectable or inoperable stage III NSCLC represents about 20–30% of all newly diagnosed NSCLC and has a 5-year survival of 35% [1]. The PACIFIC trial, a landmark study in the treatment of inoperable stage III NSCLC, demonstrated, for the first time, a survival benefit with an immune checkpoint inhibitor (ICI) in the consolidation setting. Durvalumab, an anti-programmed death ligand 1 (PD-L1) ICI, when given to patients without disease progression after definitive concurrent (cCRT), yielded unprecedented increases in progression-free survival (PFS) over placebo which ultimately translated into an overall survival (OS) benefit. The success of the PACIFIC trial provided clinical proof that the incorporation of immunomodulation with radiation therapy approaches yields meaningful clinical outcomes in stage III disease. 

The emergence of immunotherapy in the treatment paradigm of inoperable stage III NSCLC ushered in an era of accelerated knowledge of the immunomodulatory effects of radiation therapy (RT). It is now known that radiation therapy exhibits dual immunomodulatory effects that shape anti-tumor immune responses. Preclinical studies demonstrate that RT enhances CD8+ T cell activity by upregulating MHC class I expression on tumor cells, promoting dendritic cell maturation, and releasing tumor antigens and damage-associated molecular patterns (DAMPs) that prime cytotoxic T-lymphocytes [1,2]. These mechanisms foster immunogenic cell death and systemic immune activation. This was demonstrated in murine models where hypo-fractionated RT (e.g., 8 Gy × 3 fractions) amplified interferon-I (IFN-I) signaling and STING pathway activation, driving T-cell-mediated tumor control [3]. Chemotherapy (CT) synergizes with RT by further stressing tumor cells, enhancing antigen release, and sensitizing tumors to immune recognition. On the other hand, RT can also have immunosuppressive effects by recruiting regulatory T cells (Tregs), tumor-associated macrophages (M2 phenotype), and myeloid-derived suppressor cells (MDSCs), which dampen anti-tumor immunity [4]. Additionally, RT upregulates PD-L1 expression on tumor cells, creating an adaptive resistance mechanism that limits T-cell efficacy [5].

Therefore, the combination of radiotherapy with immunotherapy was a rational approach to harnessing the anti-tumor response and overcoming the immunosuppressive barriers imposed by RT. This was effective in preclinical models, in which the addition of programmed death-1 (PD-1)/PD-L1 inhibitors to RT reshaped the tumor microenvironment, boosting MHC-II+ dendritic cells and CD8+ T-cell infiltration while reducing Tregs and immunosuppressive cytokines [6]. Clinically, the PACIFIC trial became the first to demonstrate a survival benefit with such a strategy. Yet, about 50% of patients with locally advanced NSCLC will still develop disease recurrence despite consolidation durvalumab, and only about 35% will be alive in 5 years [7]. These statistics serve as the impetus to improve the outcomes beyond what has been accomplished in the PACIFIC trial.

In this narrative review, we discuss the evidence supporting the addition of immunomodulatory approaches to chemotherapy and radiation therapy in unresectable locally advanced NSCLC, provide an overview of present strategies and the prevailing challenges in the field, and offer perspectives on future approaches to enhance the efficacy of immune-based approaches for the treatment of inoperable early-stage NSCLC without actionable genomic alterations.

## 2. The Current Standard-of-Care Treatment of Inoperable Stage III NSCLC

Today’s standard of care for the treatment of patients with inoperable stage III NSCLC is chemotherapy administered concurrently with radiation therapy, followed by durvalumab for up to a year for those who do not experience disease progression after definitive cCRT. This regimen was established in the landmark PACIFIC trial, a phase 3, randomized, double-blind study that assessed the safety and efficacy of consolidation durvalumab in patients without progression after cCRT. Consolidation durvalumab significantly improved PFS [HR 0.55; 95% CI, 0.45 to 0.68; median, 16.9 v 5.6 months] and OS [stratified HR, 0.72; 95% CI, 0.59 to 0.89; median, 47.5 v 29.1 months] over placebo [7]. Consequently, durvalumab became the first FDA-approved consolidation immune checkpoint inhibitor for unresectable stage III NSCLC.

The results of the PACIFIC trial were validated not only in other prospective clinical trials that will be described below, but also in retrospective real-world data. The real-world data included the outcomes of patients who were on average older, with a poorer performance status, and experienced a longer interval between durvalumab initiation and the end of CRT [8]. PACIFIC-R (NCT03798535), an international, retrospective study, evaluated the efficacy of the regimen in patients who received durvalumab as part of an early-access program between September 2017 and December 2018. The co-primary endpoints were investigator-assessed real-world (rw) PFS and OS. The rwPFS was consistent with the data from the PACIFIC trial, demonstrating a longer PFS among patients who received cCRT versus sequential chemoradiation therapy (sCRT). The median PFS was 23.7 versus 19.3 months, respectively. Additionally, patients with PD-L1 expression greater than or equal to 1% achieved a longer rwPFS than those with PD-L1 expression of less than 1% (22.4 vs. 15.6 months) [9,10]. Similar to the PACIFIC-R study, the S-REAL study assessed the efficacy of the regimen in a multicenter, observational, retrospective study focused on a Spanish patient population who were treated as part of an early-access program between September 2017 and December 2018. In this real-world analysis, the median PFS was 16.7 months [95% CI 12.2–25], consistent with the results of PACIFIC and PACIFIC-R. The 5-year OS and PFS rates for durvalumab vs. placebo were 42.9% [95% CI, 38.2 to 47.4] vs. 33.4% [95% CI, 27.3 to 39.6] and 33.1% [95% CI 28.0 to 38.2] vs. 19.0% [95% CI 13.6 to 25.2], respectively [11]. Although some real-world studies did not show a survival benefit, several other real-world data are published reporting findings consistent with favorable outcomes with consolidation durvalumab compared to placebo. Some notable factors that account for the differences in survival include the heterogenous population of patients, including patients who are, on average, older, with a poorer performance status, and with some studies including patients with oligometastatic disease [8,12,13,14,15,16,17,18].

Despite the success of consolidation durvalumab, the sobering results of the final analyses showing that approximately 67% of patients will have disease recurrence at 5 years, and that less than 50% of patients will be alive at 5 years, underscored the need to improve outcomes for patients with inoperable stage III lung cancer [19]. The results of the PACIFIC trial, PACIFIC-R, and S-REAL can be reviewed in Table 1.

## 3. Beyond the PACIFIC Regimen: Other Explored Approaches to Improve Outcomes

Not surprisingly, in an effort to increase the chances of cure, several approaches have been explored since the PACIFIC trial. Some of these strategies include evaluating other ICIs as monotherapy consolidation, dual ICI consolidation, ICI administered concurrently with cCRT and followed by consolidation ICI, induction strategies with ICI-based therapies, and the incorporation of novel immunomodulatory agents. Key clinical trials within these categories will be discussed in this section. A summary of these trials is described in Table 1.

### 3.1. Definitive Chemoradiation Therapy Followed by Immune Checkpoint Inhibitor Monotherapy

Given the success with durvalumab, an anti-PDL1 ICI, the efficacy of monoclonal antibodies against other inhibitory checkpoint molecules such as anti-PD1 have been investigated. The LUN14-179 study was a phase 2 trial that evaluated consolidation pembrolizumab, an anti-PD1 antibody, for up to a year in patients without disease progression after definitive cCRT. The primary endpoint was time to metastatic disease or death, which was 30.7 months. The median PFS and OS were 18.7 months and 35.8 months, respectively, indicating that inhibitors against other immune checkpoints could also be of benefit over cCRT alone [20].

In the phase 3 randomized clinical trial GEMSTONE 301, patients without disease progression after definitive sCRT or cCRT were randomized to receive consolidation sugemalimab (anti-PDL1) vs. placebo. The median PFS was 9 months [95% CI 8.1–14.1] vs. 5.8 months [95% CI 4.2–6.6], respectively. The stratified hazard ratio was 0.64 [95% CI 0.48–0.85], *p* = 0.0026). These results suggested the efficacy of sugemalimab over placebo in both the cCRT and sCRT arms. It is interesting to note in this study that the 2-year duration of consolidation ICI therapy did not appear to have an improved efficacy over the standard 1-year consolidation regimens [21]. The GEMSTONE 301 study also evaluated the role of consolidation ICI after sCRT. Frail patients, or patients with a borderline performance status (PS), may not be eligible for definitive cCRT regimens due to the intensity and poor tolerability and thus rely on less intensive regimens such as sCRT in which chemotherapy is administered sequentially with RT, rather than concurrently. The PACIFIC trial excluded patients with a PS greater that 1 and did not assess the efficacy of durvalumab for patients with an ECOG of 2 or those receiving sCRT. Subsequently, efforts were made to understand the role of consolidation ICI therapy following sCRT.

PACIFIC 6, a phase 2 multicenter, open-label, single-arm trial, evaluated consolidation durvalumab in patients who were not suitable for cCRT and did not have disease progression after sCRT. About 60% of patients had a PS of 1 or greater and had stage IIIB or IIIC disease. The median PFS was 10.9 months [95% CI 7.3–15.6], while the 12-month PFS was 49.6%. The safety results were consistent with those of the PACIFIC trial. Overall, these results were practice-informing and showed that consolidation durvalumab could be safely administered to patients with a less robust performance status after sCRT while sustaining the clinical benefits seen in the PACIFIC trial [22]. Similarly, the PACIFIC 5 trial, a phase 3 study, randomized patients without disease progression following definitive cCRT or sCRT to receive consolidation durvalumab or placebo. In this study, a predominantly Asian patient population was enrolled (71.9%). As expected, an improvement in PFS was demonstrated with durvalumab over placebo (HR 0.75; 95% CI: 0.58–0.99; *p* = 0.038). The median PFS was 14.0 (95% CI:10.9–18.0) vs. 6.5 (95% CI: 5.4–13.8) months. A benefit was seen regardless of the CRT modality; cCRT (HR 0.76; 95% CI: 0.55–1.06) or sCRT (HR 0.75; 95% CI: 0.49–1.18) [23].

### 3.2. Definitive Chemoradiation Followed by Dual Immunomodulator Consolidation

Several efforts have been made to evaluate the incremental benefit of dual ICI consolidation. BTCRC-LUN16-081, a randomized phase 2 multicenter trial, compared consolidation nivolumab (anti-PD1) to dual nivolumab plus ipilimumab (anti-CTLA4) in stage IIIA/IIIB patients without disease progression after cCRT. Dual ICI consolidation was continued for up to 24 weeks (6 months). No significant improvement in efficacy outcomes was seen in the nivolumab plus ipilimumab arm when compared to the nivolumab-alone arm. The 18-month PFS was 62.3% vs. 67% (*p* < 0.1) with nivolumab vs. nivolumab plus ipilimumab. The median PFS was 25.8 months (95% CI: 16.5 months-NR) with nivolumab arm vs. 25.4 months with nivolumab plus ipilimumab (95% CI 18.6 months-NR). The 18- and 24-month OS estimates were 82.1% and 76.6% with single-agent nivolumab compared to 85.5% and 82.8% with combination nivolumab plus ipilimumab. Moreover, safety was a concern in the dual ICI arm grade-3-or-greater treatment-related adverse events (trAEs) were higher with the combination treatment (15.7%) compared to single-agent nivolumab (9.3%) [24]. The higher incidence of trAEs without significant improvement in efficacy argues against the use of combination nivolumab plus ipilimumab for consolidation in this setting.

The COAST trial also investigated dual immunomodulatory consolidation approaches. This open-label phase 2 platform investigated consolidation durvalumab monotherapy, durvalumab plus oleclumab (anti-CD73), and durvalumab plus monalizumab (anti-NKG2A). Interesting, the results of this study suggest the improved efficacy of the combination approaches compared to durvalumab monotherapy. The overall response rate (ORR) was 30.0% (95% CI, 18.8 to 43.2), 35.5% (95% CI, 23.7 to 48.7), and 17.9% (95% CI, 9.6 to 29.2) in the durvalumab plus oleclumab, durvalumab plus monalizumab, and durvalumab-alone arms, respectively. Similarly, the combination arms performed better than durvalumab for PFS with an HR of 0.44 [95% CI, 0.26 to 0.75] favoring the durvalumab plus oleclumab arm, and an HR of 0.42 [95% CI, 0.24 to 0.72], in favor of the durvalumab plus monalizumab arm. Additionally, the 12-month PFS rates appeared to be higher in the combination consolidation arms at 62.6% [95% CI, 48.1 to 74.2] with durvalumab plus oleclumab, 72.7% [95% CI, 58.8 to 82.6] with durvalumab plus monalizumab, and 33.9% [95% CI, 21.2 to 47.1] with durvalumab alone. These findings are currently under investigation in the ongoing Phase 3 study of durvalumab combined with oleclumab or monalizumab in patients with unresectable stage III NSCLC (PACIFIC 9) [25].

The promising results of the COAST platform present an opportunity to evaluate other combination immunomodulatory approaches for consolidation therapy in patients whose cancers did not progress after definitive chemoradiation therapy. A focused effort on mechanistically synergistic immunomodulatory combinatorial approaches may begin to pave the way for future success in this disease stage. In addition to PACIFIC 9, the ongoing phase 3 SKYSCRAPER-03 study, comparing combination atezolizumab (anti-PDL1) plus tiragolumab (anti-TIGIT) to durvalumab consolidation after cCRT and will yield additional results for the consideration of dual consolidation immunomodulatory approaches [26,27].

Dual immunotherapy consolidation has also been investigated after concurrent administration of ICI therapy during cCRT and will be discussed in the section below.

### 3.3. Immunotherapy Administered Concurrently with cCRT Followed by Consolidation Checkpoint Blockade

The rationale for concurrent administration of immunotherapy with definitive cCRT stemmed from preclinical models and retrospective analyses that suggested synergy between radiation therapy and anti-PD1 therapies [5,28,29,30]. Additionally, the improved outcomes of concurrent chemoimmunotherapy in metastatic NSCLC suggested that this approach was rational [31]. However, the results of the studies with concurrently administered ICI and CRT were underwhelming.

PACIFIC 2 attempted to improve upon the outcomes of the PACIFIC trial with the use of concurrent administration of durvalumab and cCRT. This was a phase 3 randomized double-blinded international study that compared concurrent durvalumab and cCRT vs. concurrent placebo and cCRT followed by consolidation durvalumab or placebo in patients with unresectable stage III NSCLC. Concurrently administered durvalumab and cCRT followed by durvalumab did not statistically improve PFS or OS when compared to the placebo arm (HR, 0.85; 95% CI: 0.65–1.12; *p* = 0.247). The median PFS was 13.8 vs. 9.4 months in the durvalumab vs. placebo arms. Additionally, there was no OS benefit with the addition of durvalumab concurrently to cCRT and consolidation, compared to placebo. The median OS was 36.4 vs. 29.5 months (HR, 1.03; 95% CI: 0.78–1.39; *p* = 0.823). Of note, in the first 4 months of combination treatment with immunotherapy and cCRT, there were a higher number of trAEs leading to treatment discontinuation (14.2% vs. 5.6%) or death (6.8% vs. 4.6%) in the durvalumab arm [32]. At first, the result of PACIFIC 2 was surprising in view of the success of the PACIFIC trial; however, the negative results are consistent with other clinical investigations with concurrently administered ICI + cCRT.

The NICOLAS trial evaluated nivolumab and cCRT followed by 1 year of consolidation with nivolumab. The 1-year PFS was the primary endpoint and resulted in a value of 53.7% (95% CI: 42.0–64.0%). At 3 years of follow up, the median OS was 38.8 months [33]. The trial did not meet its primary endpoint of a 15% improvement in 1-year PFS compared to the PACIFIC historical control of 45% [33]. The DETERRED trial was a phase 2 study which consisted of two parts. Part 1 compared the safety and efficacy of concurrent atezolizumab and cCRT followed by consolidation atezolizumab, and Part 2 evaluated definitive cCRT alone followed by atezolizumab consolidation. The median PFS for cCRT followed by consolidation atezolizumab was 18.9 months, while the median PFS for atezolizumab + cCRT followed by consolidation atezolizumab was 15.1 months. Conversely, the median OS for the consolidation-only arm was 26.5 months and was not reached for the concurrent arm [34].

Concurrently administered ICI + cCRT has also been investigated with dual ICI consolidation. Checkmate 73L (CM73L), an international phase 3 study, randomized patients 1:1:1 to receive nivolumab plus cCRT followed by dual consolidation nivolumab and ipilimumab vs. nivolumab plus cCRT followed by nivolumab alone vs. standard-of-care cCRT followed by durvalumab for up to 1 year. The median PFS in the concurrent nivolumab and cCRT followed by nivolumab and ipilimumab was 16.7 months compared to 15.6 months in the SOC arm (HR 0.95 [96% CI, 0.77–1.19]; *p* = 0.6461). A comparison of OS in the concurrent nivolumab and cCRT followed by nivolumab and ipilimumab arm vs. the standard-of-care arm produced an HR of 1.12 (95% CI, 0.87–1.43). When compared to the standard-of-care arm, the PFS and OS for the concurrent nivolumab and cCRT arm were 0.84 [0.69–1.04] and 0.97 [0.76–1.24], respectively [35]. Similar to BTCRC-LUN16-08, these results ruled against dual consolidation with ipilimumab and nivolumab. Moreover, adding immune checkpoint blockade to cCRT was not of incremental benefit to consolidation ICI therapy.

The CHORUS trial also investigated dual immunotherapy consolidation after definitive cCRT + immunomodulatory therapy. This was a phase 1/2 trial that evaluated the efficacy of definitive cCRT in combination with canakinumab (anti-IL1β antibody) followed by dual durvalumab and canakinumab consolidation in 32 patients with stage III NSCLC. The primary outcome of PFS is still immature, but the ORR at an interim analysis at the end of concurrent canakinumab and cCRT was 72%. Twenty-five percent of patients demonstrated stable disease (SD), but one patient was non-evaluable. There was no progressive disease in this patient population at the end of cCRT and canakinumab. At 11.2 months of follow-up, 26 patients (81%) had a complete or partial response as the best overall response to therapy and eight patients had confirmed progressive disease. An interesting aspect of this study was the use of circulating tumor DNA (ctDNA) testing. In the initial report, a direct correlation was made between ctDNA detection and the best responses. In total, 100% of patients (10) who had undetectable ctDNA 3 weeks into treatment had a partial response or complete response on initial imaging compared to 60% of patients with detectable ctDNA. Amongst patients with undetectable ctDNA at 16 weeks post-treatment, 90% (18) had a partial or complete response, while 67% of those with detectable ctDNA had PR/CR. Of note, all patients with detectable ctDNA and stable disease as the best response subsequently developed progressive disease. It would be interesting to know whether there were any patients with SD who had undetectable ctDNA. Could it be that SD would also predict disease progression and not just ctDNA detection [36]?

The final analysis of the phase 2 Keynote-799 was recently reported. The study investigated two cohorts. Cohort A (squamous and non-squamous) was given one cycle of carboplatin/paclitaxel/pembrolizumab followed by two cycles carboplatin/paclitaxel/pembrolizumab concurrently with RT. Cohort B (non-squamous) received three cycles of cisplatin, pemetrexed, and pembrolizumab concurrently with RT. In both cohorts, patients who did not experience disease progression after cCRT were treated with consolidation pembrolizumab for up to a year. The ORR was 71% (95% CI 62.1–79.6) for cohort A and 75.5% (95% CI 66.0 and 83.5) for cohort B. The median PFS was 29.0 (16.6–48.5) for cohort A and 45.3 (17.9-NR) for cohort B. The median OS was 35.6 months (26.1–44.2) for cohort A and 56.7 (41.1-NR) for cohort B [37,38].

Taken together, most of these results suggest a lack of incremental benefit to the addition of immune checkpoint blockade concurrently with cCRT. The KEYLYNK012 trial is confirming the findings of Keynote 799 in a phase 3 study and will provide additional insight when the results are reported [39]; however, there is now evidence that the lack of benefit when immune checkpoint inhibition is administered concurrently with cCRT could be due to the detrimental effect of direct radiation on T cells, limiting their activity [40]. This suggests that checkpoint blockade could be best reserved at treatment times outside of radiation administration when a robust immune response can be elicited in the presence of neoantigens by the tumor cells and/or radiation killing of the tumor cells. It is possible that concurrent administration of a different class of immunomodulators, such as the use of canakinumab as in the CHORUS trial, could be beneficial, but the final result of that study is pending. Further research with the concurrent administration of other agents will be necessary to determine the utility of additional agents concurrently with RT.

### 3.4. Induction Immune Checkpoint Blockade Followed by CCRT and Consolidation Checkpoint Blockade Strategies

Induction therapy has become the mainstay of therapy in early-stage surgically resectable NSCLC; however, its incorporation into unresectable stage III disease has lagged behind. The benefits of induction therapy include early treatment of micro-metastatic disease [41]. Moreover, the ability to harness the immune system prior to the detrimental effect of radiation therapy offers an additional benefit to the induction approach [42,43,44]. The phase 2 AFT-16 trial evaluated up to four cycles of induction atezolizumab, followed by cCRT and 1 year of consolidation atezolizumab and showed promising results. The primary endpoint was the disease control rate (DCR) at 12 weeks. There were 30.7% partial responders and 46.8% with stable disease, making for a DCR of 77.4% (80% CI, 69.2–84.3%). DCR was 82.4% vs. 90.9% in patients with PD-L1-negative or -positive tumors, respectively. TrAEs were reported in 54 of 62 patients (87.1%), most of which were grade 1. The 12-month PFS from the start of induction atezolizumab was 66% (95% CI 55–79), while the 18-month PFS from the same time point was 57% (95% CI, 45–71). The median PFS was 23.7 (95% CI, 13.2-NE). In the most recent analysis, the 18-month OS was 84% (95% CI 75–94) [45].

Several other ongoing studies are now investigating this approach. Their findings will provide additional guidance for new studies investigating novel approaches.

### 3.5. Agents That Potentiate Immunotherapy Response, Ongoing Trials of Potentiating Agents

The papers summarized above have illustrated the promise of ICIs for patients with unresectable stage III NSCLC. The disappointing results of several trials have demonstrated the need to expand the success of ICI consolidation to a broader range of immunomodulatory agents. Many investigational approaches are underway, but agents that potentiate an immune response have yet to gather momentum in the care of patients with stage III NSCLC. The mechanisms outlined below are by no means exhaustive but do provide a framework for some of the best-investigated agents and pathways for the potentiation of immunotherapeutic agents.

The adenosine pathway has become a target for many malignancies treated with immunotherapies. Adenosine is an immunosuppressive metabolite within the tumor microenvironment, contributing to tumor growth, invasion, and immune evasion. It is produced at high levels in hypoxic conditions typical of tumors, where it suppresses immune responses through its actions on T- and NK-cell function via its actions on the purine signaling axis. Adenosine signaling dampens the immune response, thereby promoting tumor survival and metastasis primarily through the CD39/73 axis, though alternative pathways are also present and contribute to the immunosuppressive tumor microenvironment [46,47,48]. Preclinical studies have shown that adenosine receptor antagonists can potentiate the effects of existing immunotherapies [49,50]. This was investigated in the COAST platform, which studied the use of durvalumab alone or in combination with oleclumab or monalizumab in patients with unresectable stage III NSCLC. In the COAST study, olecumab, an anti-CD73, which exerts its actions via inhibition of the adenosine pathway, was shown to improve ORR and PFS when used in combination with consolidation durvalumab. Of note, in the COAST study, consolidation durvalumab and monalizumab (a humanized anti-NKG2A antibody that reduces the inhibition of T and natural killer cells) also showed a superior ORR and PFS when compared to consolidation durvalumab alone [25].

Targeting other inhibitory receptors has long been an alluring path forward for improving outcomes for patients treated with immunotherapeutic agents. One such method is through inhibition of the T cell immunoglobulin and ITIM domain (TIGIT), a co-inhibitory immune checkpoint receptor whose inhibition reduces the hinderance of T and NK cells in the tumor microenvironment. Preliminary results of ACR-7 demonstrated that a blockade of TIGIT in combination with PD-1 inhibition improved survival outcomes in stage IV NSCLC [51]. PACIFIC 8 is a phase 3, double-blind, placebo-controlled, randomized, global trial, with the aim of proving the concept in patients with unresectable stage III disease. In this trial, patients with PD-L1 positive, unresectable stage III NSCLC who did not progress on cCRT will be randomized to receive standard-of-care durvalumab with domvanalimab (anti-TIGIT) or a placebo [52]. Another trial, NCT05798663, is a phase 2 trial investigating the use of neoadjuvant and adjuvant atezolizumab with or without tiragolumab (anti-TIGIT) with chemoradiotherapy for unresectable stage III NSCLC. Results from a similar study, SKYSCRAPER-03 (NCT04513925), will compare atezolizumab and tiragolumab with durvalumab in patients with locally advanced, unresectable stage III NSCLC who have not progressed after concurrent platinum-based chemoradiation.

Targeting co-stimulatory molecules is a similarly appealing approach for potentiating a response to immunotherapy. Targeting OX40 (CD134) has recently been identified as a feasible path. OX40 is expressed on T cells and enhances their activation, signaling, proliferation, and survival [53,54,55]. Preclinical models have shown promising results [56]. NCT06623136 is an upcoming study of the combination of Toripalimab (an ICI) in combination with ES102 (an OX40 agonist) for patients with unresectable locally advanced and metastatic non-small cell lung cancer not suitable for radical concurrent chemoradiotherapy and PD-L1 TPS ≥ 50%. NCT04198766 is a phase 1/2, open-label, non-randomized, four-part trial, investigating INBRX-106 (an OX40 agonist) in combination with pembrolizumab. The trial will enroll patients with NSCLC (and other cancer types) that has progressed despite all standard therapies including ICI during Parts 2 and 4.

PCSK9, a protein known for its role in cholesterol metabolism, has recently been recognized for its negative impact on the efficacy of ICIs. High expression of PCSK9 in tumor tissues is associated with reduced lymphocyte infiltration and poorer outcomes in patients undergoing therapy with an ICI. Inhibition of PCSK9 leads to increased expression and decreased degradation of major histocompatibility complex class I (MHC I) proteins on tumor cells, leading to increased T cell infiltration of the tumor and improved anti-tumor activity [57,58,59]. TOP 2201 (NCT05553834) is a phase 2 trial of the PCSK9 inhibitor Alirocumab and the PD-1 inhibitor Cemiplimab in patients with metastatic NSCLC refractory to previous treatment with an anti-PD-1 agent. No preliminary data are available for TOP 2201 at this time. TOP2301 is also investigating a combination of neoadjuvant anti-PCSK9 and chemoimmunotherapy in surgically resectable NSCLC.

The phosphatidylinositol-3-kinase (PI3K) pathway and histone deacetylases (HDACs) have long been known to be crucial to tumor cell survival and proliferation. Inhibition of the PI3K pathway disrupts tumor growth and makes tumors more susceptible to attack from immune cells by altering the tumor microenvironment. PI3K inhibitors also reduce the suppressive activity of myeloid cells that contribute to ICI resistance [60,61,62]. HDAC inhibitors enhance the effects of ICI by making cancer cells more recognizable to the immune system through the alteration of gene expression [63,64]. The inhibition of PI3K and HDACs induces ferroptosis and stimulates an inflammatory tumor microenvironment, reducing immune suppression and potentially increasing response to ICIs [64].

Antiangiogenic agents have also been investigated in combination with ICIs. These agents, which target VEGF and ANG2, enhance ICIs by normalizing the tumor microenvironment, improving immune cell infiltration, and facilitating an immunosupportive state [65,66,67]. Although the combination of antiangiogenic agents and ICIs has been investigated in advanced NSCLC, none of these studies included standard-of-care cCRT, and there are no studies of antiangiogenic agents with adjuvant ICIs after CCRT [68,69,70]. In fact, despite the potential for radio-sensitization, antiangiogenic agents such as bevacizumab have notably been associated with severe hemorrhagic toxicities when combined with thoracic radiation, limiting their use in curative-intent multi-modal regimens for NSCLC patients [71].

Anti-PD-L1 fusion proteins are also under investigation. NCT05177497 is a phase 2, open-label, single arm study investigating SHR-1701, a novel bifunctional fusion protein composed of a mAb against PD-L1 fused with the extracellular domain of TGF-β receptor II, as a consolidation strategy in inoperable stage III NSCLC.

Others have studied the use of oncoviruses to stimulate an immunosupportive tumor microenvironment. NCT04495153 is a study of CAN-2409, a replication-defective adenoviral construct encoding the herpes simplex virus thymidine kinase (HSV-tk) gene that is injected intratumorally followed by 2 weeks of prodrug administration (valacyclovir or acyclovir). In this study, CAN-2409 was injected intratumorally to patients with unresectable stage III or stage IV NSCLC in combination with a standard-of-care ICI. At this time, the study is not recruiting, but no data are available for review [72].

Nanoparticle (NP) development is a new approach in which NPs serve as vehicles to improve the pharmacodynamics of previously undeliverable agents. NP-based approaches boast not only of improved delivery of agents, but also reductions in the off-target effects of immunotherapeutic agents, potentially improving both efficacy and tolerability [73]. NCT06667908 is an active clinical trial currently studying the combination of JNJ-90301900 with CCRT followed by consolidative immunotherapy.

Finally, other studies are focusing their investigations on the combination of ICIs with other known chemotherapeutics, including olaparib (NCT04380636), vinorelbine (NCT06540950), and antibody–drug conjugates such as Datopotamab Deruxtecan (NCT05687266). The outcomes of these studies are currently unknown, but their potential to drive the field forward is encouraging and gives hope to patients with unresectable stage III NSCLC. Table 2 summarizes the trials discussed in this section amongst other notable ongoing trials in this space.

## 4. Current Challenges and Future Directions

Tremendous effort has been put toward improving the outcomes of patients with unresectable Stage III NSCLC. However, to date, no clinical trial has demonstrated superior results over the PACIFIC trial in a phase 3 study. Thus, the 5-year OS remains below 50%. The preclinical and clinical research efforts already undertaken and/or currently under investigation have provided insights to some prevailing questions in the field.

### 4.1. Sequencing of Treatment

A crucial question regards the optimal sequencing of checkpoint inhibition (induction vs. concurrent vs. consolidation-only checkpoint inhibition). The answer to this question is guided by the results of two phase 3 clinical trials in which head-to-head comparisons of concurrent ICI + cCRT vs. ICI consolidation were assessed: the PACIFIC 2 study and Checkmate 73L.

In Checkmate 73L, described above, concurrently administered nivolumab and cCRT followed by either dual consolidation with nivolumab and ipilimumab or nivolumab alone did not show significant improvements in PFS or OS over the standard-of-care cCRT followed by consolidation durvalumab. Similarly, in PACIFIC 2, concurrent durvalumab and cCRT followed by durvalumab consolidation showed no improvement in outcomes over cCRT alone followed by placebo consolidation. Although the comparator arm of the PACIFIC 2 study does not directly compare with the PACIFIC study, the comparator arm of both studies was treated similarly. Given the success of the consolidation durvalumab alone in the PACIFIC study, the lack of benefit in the PACIFIC 2 study was surprising. Further evidence arguing against the concurrent administration of ICI with cCRT was demonstrated in the DETERRED trial. Paradoxically, cCRT followed by atezolizumab numerically outperformed concurrent cCRT and atezolizumab followed by a year of atezolizumab consolidation. The results of the Keynote 799 [37] and the KEYLYNK012 [39] studies are keenly awaited; however, recent understanding of the negative impact of cCRT on T cells may explain the lack of benefit seen when ICIs are administered concurrently with RT.

The success of neoadjuvant therapies in surgically resectable NSCLC has paved the way for induction ICI approaches followed by definitive cCRT and a consolidation checkpoint blockade as a promising strategy. Although the results of the AFT-16 trial were modest, the ongoing studies will likely provide additional clinical data to further evaluate this strategy in unresectable stage III NSCLC.

### 4.2. Duration of Consolidation

The PACIFIC trial utilized one year of consolidation durvalumab in patients without disease progression after concurrent chemotherapy. Although the choice regarding the duration of one year was arbitrary, up to one year of consolidation durvalumab became the standard of care for patients without disease progression at the end of definitive cCRT. It is fair to say that the optimal duration of consolidation immunotherapy is not known. There are limited data on trials that have investigated other durations of consolidation ICI after definitive cCRT. In the phase 3 GEMSTONE trial, the anti-PDL1 agent sugemalimab was used as consolidative therapy for two years after sCRT. The median 1-year PFS for sugemalimab does not provide any evidence to favor prolonged consolidation ICI therapy. A shorter duration was tested in the BTCRC-LUN 16-081 trial, where dual consolidation with ipilimumab plus nivolumab for 6 months was compared with consolidation nivolumab alone for 6 months. As described previously, there were no significant differences in efficacy outcomes between the two arms. Similar to the GEMSTONE trial, the efficacy results do not provide data to justify the de-escalation of treatment duration.

Indeed, the question regarding the optimal duration of therapy for checkpoint inhibitors is not unique to unresectable stage III NSCLC. In metastatic disease, maintenance of the immune checkpoint blockade is continued for up to 24 months, mostly as a result of clinical trial design [34,74]. In one multicenter retrospective analysis, the clinical outcomes of 96 metastatic NSCLC patients who completed two years of ICI were evaluated. The 12-month PFS and OS rates for patients who completed two years of ICI were 81% and 96.4%, respectively. Amongst patients who discontinued ICI early either due to trAEs or other reasons, the 12-month PFS and OS rates were 71% and 90%, respectively [75]. Similarly, another retrospective analysis was performed in 52 metastatic melanoma patients who received anti-PD1 for a median of 11 months. Complete response was achieved in 25% of patients, while 54% and 21% had a partial response and stable disease, respectively. At a median follow up of 20.5 months (range 3–49), 25% of patients had disease progression; however, 75% of patients remained without disease progression [76]. These results collectively indicate durable responses in patients treated with ICI who respond to therapy, but leave open the question of whether less or more than a year or two of ICI therapy is sufficient or necessary.

While insight has been gained into the durable responses to ICI, there are insufficient prospective data to guide the choice of 6, 12, or 24 months of consolidation. Two ongoing studies on unresectable stage III NSCLC utilizing two years of consolidation ICI will provide additional guidance. One study (NCT03379441) is investigating two years of maintenance pembrolizumab in unresectable stage IIIA and IIIB NSCLC after definitive cCRT/sCRT [77]. The BRIDGE trial is investigating two years of consolidation durvalumab after induction durvalumab + chemotherapy and concurrent durvalumab + RT [78].

Thus, consolidation immune checkpoint blockade for up to one year remains the standard of care after definitive cCRT in unresectable stage III NSCLC.

### 4.3. Tolerability

Tolerability should be taken into consideration in individual patients when determining treatment modality and duration, with the goal of limiting toxicity while improving efficacy. This is particularly important with elderly and frail patients. A retrospective analysis conducted between September 2017 and September 2022 showed that elderly patients ≥70 years old were less likely to complete durvalumab compared to those <70 years (27.5% vs. 39.2%; *p* = 0.040). Elderly patients also experienced more trAEs, grade 3/4 trAEs, permanent discontinuation of durvalumab, and treatment-related deaths. Pulmonary AE in elderly patients significantly led to treatment discontinuation or death [79].

In elderly and frail patients, sCRT is often chosen as the radiation modality of choice due to better tolerability in patients with suboptimal PS. Since the PACIFIC trial evaluated consolidation durvalumab after CCRT, the question was also raised regarding the impact of consolidation immunotherapy after sCRT for patients who are unable to tolerate cCRT. A few studies have provided an answer to this question. Consolidation immune checkpoint blockade showed a benefit over a placebo regardless of whether patients received cCRT or sCRT [21,22,23]. The PACIFIC 5, PACIFIC 6, and GEMSTONE studies unequivocally showed the benefit of consolidation immunotherapy in patients who received either cCRT or sCRT. In the PACIFIC 5 study, there was a numerical benefit in patients who received cCRT compared to sCRT, which suggests that cCRT should be considered the CRT of choice in patients who can tolerate it. In the PACIFIC-R, the rwPFS was also numerically longer among patients who received concurrent versus sequential CRT (median, 23.7 versus 19.3 months). Consistently, in the S-REAL study, rwPFS was higher in patients who had received prior cCRT versus sCRT (20.6 versus 9.4 months). While sCRT appears to be an inferior strategy that should be reserved for patients who cannot tolerate cCRT, there is now clearer evidence to support the use of consolidation ICI therapy after sCRT. Therefore, the use of sCRT should not preclude patients from the benefit of consolidation durvalumab.

Further, de-escalation regimens have been investigated. The SPRINT trial is a phase 2 study that evaluated a chemotherapy free approach in patients with PDL1 > 50% [80]. Patients received induction pembrolizumab for three cycles followed by a risk-adapted hypofractionated RT regimen based on metabolic tumor volume and one year of consolidation pembrolizumab. The data showed promising results that this approach is feasible, safe, and efficacious. The 1-year PFS was 76%. OS rates were 92% and 76% at 1 and 2 years, respectively. No grade 4–5 adverse events were reported. Another de-escalation study is a phase 2 study of single-agent carboplatin plus radiation followed by durvalumab in patients with stage III NSCLC with a PS of 2 (NEJ039A trial) [81]. The TRADE study is another phase 2 clinical trial investigating a chemotherapy-free regimen in frail patients. Patients will receive durvalumab in combination with either conventionally fractionated or hypofractionated thoracic radiotherapy [82].

### 4.4. Dose and Fraction of Radiation Therapy

The treatment of stage III NSCLC has significantly evolved over the decades, starting with advancements in definitive CRT. Initial studies, such as RTOG 7301, established a standard dose of 60 Gy for definitive RT, which laid the groundwork for subsequent research [83]. The CALGB 8433 and RTOG 8808 trials highlighted the advantages of combining sequential chemotherapy with RT compared to RT alone, leading to improved survival outcomes [84,85]. Further exploration in RTOG 9410 indicated that concurrent chemotherapy combined with RT offered superior benefits over sequential approaches, a finding supported by a meta-analysis that reinforced the survival advantage of concurrent chemoradiation [86,87].

A significant proportion of patients enrolled in the PACIFIC trial were deemed ineligible for randomization following concurrent cCRT due to disease progression, highlighting a critical area for improvement in treatment approaches before the initiation of consolidation ICI. This observation underscores the importance of optimizing the concurrent CRT strategy to ensure that a larger cohort of patients can benefit from subsequent immunotherapeutic interventions. The RTOG 0617 trial played a crucial role in shaping the current understanding of radiation therapy dosing in stage III NSCLC. While earlier phase 2 studies had suggested potential benefits from escalating the radiation dose beyond 60 Gy, the results from RTOG 0617 directly challenged this notion by demonstrating that increasing the dose did not lead to improved survival outcomes. In fact, the trial revealed that patients receiving higher doses of radiation therapy had worse outcomes, likely due to treatment-related toxicity related to a multitude of factors [88]. More recent studies, such as the randomized phase 3 NARLAL2 trial, have demonstrated that isotoxic RT dose escalation (FDG-PET-guided in this case) for locally advanced NSCLC is possible, with efficacy data pending [89]. This is particularly important and relevant in the immunotherapy era, given that emerging evidence suggests that the dose and fractionation of tumor-directed radiation therapy play a pivotal role in shaping the tumor microenvironment and may significantly influence the induction of an effective anti-tumor immune response. Preclinical studies have indicated that higher, ablative doses of radiation can not only result in improved locoregional control but can also create a more immunogenic environment, potentially enhancing the efficacy of immune checkpoint inhibitors [6]. The effects of RT dose and fractionation on the tumor immune microenvironment (TME) are increasingly recognized as critical factors in shaping anti-tumor immune responses. Hypofractionated regimens have emerged as particularly immunogenic, promoting dendritic cell maturation and immunogenic cell death (ICD) through the release of DAMPs [90]. To this end, ongoing studies such as NRG-LU008 are investigating the treatment paradigm of Stereotactic Body Radiation Therapy (SBRT) in a primary lung tumor followed by CRT to nodal disease, followed by consolidation ICI. Optimizing the RT dose, fractionation, and delivery timing remains pivotal for balancing immune activation.

### 4.5. Biomarker Selection

The importance of biomarker selection for treatment approaches in NSCLC is increasingly recognized. Yet, the development of biomarkers of response, or lack thereof, is challenging and often lags behind the clinical development of novel treatment modalities. The PACIFIC trial did not select for biomarkers and included patients irrespective of genomic status as there was no other established alternative standard of care for those patients at the time of study conception. Although the numbers were small, subgroup analysis raised the suspicion that patients with EGFR mutation lacked significant benefit from this modality. It is now established that patients with sensitizing EGFR alterations predict a lack of response to immune checkpoint inhibitors in this setting, reflected in the NCCN guidelines [91,92]. Although the role of ALK alterations is not as clearly defined, patients with driver ALK alterations are now excluded from immunotherapy clinical trials. The role of other actionable alterations is not as clear. However, clinical trials exploring consolidation therapy with targeted agents are currently underway [93].

The role of PD-L1 status in the response to consolidation durvalumab has been questioned. In the PACIFIC trial, analysis by the prespecified subgroups showed a benefit irrespective of PD-L1 status greater or less than 25%. A post hoc analysis did not show a consistent OS benefit in the PD-L1-negative patient [94]. However, the role of PD-L1 negativity remains controversial and inconsistent across different reports. In the PACIFIC-R study, the real-world PFS was 22.4 versus 15.6 months in PDL1-positive or -negative cases but this was not statistically significant [9]. Conversely, the S-REAL study did not show a remarkable difference in PFS between patients with PD-L1 ≥ 1% or < 1% (16.7 versus 15.6 months, respectively) [11].

There are emerging data regarding the use of minimal residual disease (MRD) as detected by ctDNA testing as a prognostic indicator and a modality to guide the intensification or de-escalation of consolidation therapy. The potential for MRD testing was demonstrated in the CHORUS trial. The interim results, as described previously, suggested that patients can be stratified by the presence of MRD post cCRT [36]. Recent results from Keynote 799 corroborate these findings. In the 67 patients with detectable ctDNA, the ORR was 68.7% vs. 50% in 6 patients without detectable ctDNA. Furthermore, there was a trend towards improved PFS and OS in patients who cleared their ctDNA at the seventh cycle [95]. Although intriguing, the role of ctDNA is still emerging and needs to be confirmed in other studies prior to incorporation into clinical practice for stage III disease.

Another evaluated approach is radiomics. The use of quantified radiological features, radiomics, to predict responses and disease recurrence is another area of investigation for biomarker development in stage III NSCLC [96]. The available evidence for radiomics stems from retrospective analyses in which it was demonstrated that radiological features corresponded with the response to therapy [97,98,99]. The clinical utility of this approach remains uncertain. Prospective studies are needed to guide their use.

## 5. Conclusions

The PACIFIC regimen, consisting of one year of consolidation durvalumab after concurrent CRT, is a landmark advancement in the treatment of stage III inoperable NSCLC. Despite consolidation ICI, however, many patients remain at risk of recurrence and death. Comprehensive efforts aimed at improving the outcomes of these patients have been made and several are still underway. Unfortunately, none have shown significant benefits over the PACIFIC regimen in a phase 3 trial to result in a practice-changing shift. Several studies are underway and will yield results in the next few years with the hope of moving the needle forward. The investigation of strategies to further harness the immune system continues to be an area of need in stage III disease. Future studies focusing on combination immunomodulatory approaches that are synergistic with radiotherapy and add to the benefit of consolidative checkpoint inhibition are needed. A more thorough understanding of biomarkers that predict response and resistance to these strategies and adequate patient selection is necessary to limit toxicities while maximizing outcomes.

## Figures and Tables

**Table 1:** Trials shaping the current landscape of the treatment of patients with unresectable stage III NSCLC.

Study Name (NCT)	Trial Design					Median PFS (Months)	Median OS (Months)
	Phase	CRT Type	Arms *	Duration	Primary Endpoint		
**Single Immunotherapy Consolidation**							
PACIFIC (NCT02125461)	III randomized	cCRT	Durvalumab (*n* = 473) vs. Placebo (*n* = 236)	1 year	PFS OS	17.2 vs. 5.6 HR: 0.51 CI: 0.41–0.63	47.5 vs. 29.1 HR: 0.68 ^+^ CI: 0.47–0.90
LUN14-179 (NCT02343952)	II single arm	cCRT	Pembrolizumab (*n* = 93)	1 year	TMDD	18.7	35.8
GEMSTONE-301 (NCT03728556)	III randomized	sCRT or cCRT	Sugelimumab (*n =* 255) vs. Placebo (*n* = 126)	2 years	PFS	9.0 vs. 5.8 HR: 0.64 CI: 0.48–0.85	NR ^1^ v NR
PACIFIC 6 (NCT03693300)	II single arm	sCRT	Durvalumab (*n* = 117)	1 year	G3-4 AEs	10.9 CI: 7.3–15.6	^2^
PACIFIC 5	III randomized	cCRT or sCRT	Durvalumab (*n* = 251) vs. Placebo (*n* = 128)	1 year	PFS	14.0 vs. 6.5 HR: 0.75 CI: 0.58–0.99	-
**Dual Immunotherapy Consolidation**							
BTCRC-LUN 16-081 (NCT03285321)	II randomized	cCRT	Nivolumab (*n* = 54) vs. nivolumab + ipilimumab (*n* = 51)	6 months	18-month PFS	25.8 vs. 25.4	NR ^3^ vs. NR ^4^
COAST (NCT03822351)	II randomized	cCRT	Durvalumab [D] (*n* = 66) vs. durvalumab + oleclumab [DO] (*n* = 59) Vs. durvalumab + monalizumab [DM] (*n* = 61)	1 year	IA-ORR	6.3 vs. NR vs. 15.1 DO vs. D HR: 0.44 CI: 0.26–0.75 DM vs. D HR: 0.42 CI: 0.24–0.72	-
**Concurrently Administered cCRT + Immunotherapy Followed by Consolidation**							
PACIFIC 2 (NCT03519971)	II randomized	cCRT	cCRT + durvalumab → durvalumab (*n* = 219) vs. cCRT + placebo → placebo (*n* = 108)	1 year	PFS	13.8 vs. 9.4 HR: 0.85 CI: 0.65–1.12	36.4 vs. 29.5 HR: 1.03 CI: 0.78–1.39
NICOLAS (NCT02434081)	II single arm	cCRT	cCRT + nivolumab → nivolumab (*n* = 79)	1 year	1-year PFS	12.7 ^5^ CI: 10.1–22.8	38.8 ^6^ CI: 26.8–NE
DETERRED (NCT02525757)	II 2 parts	cCRT	cCRT → atezolizumab (*n* = 10) vs. cCRT + atezolizumab → atezolizumab (*n* = 30)	1 year	Safety	18.9 vs. 15.1 HR: 1.30 CI: 0.56–3.04	26.5 vs. NR HR: 0.71 CI: 0.25–1.99
Checkmate 73L (NCT04026412)	III randomized	cCRT	A: Nivolumab + cCRT→ nivolumab + ipilimumab (*n* = 287) vs. B: Nivolumab + cCRT → nivolumab (*n* = 320) vs. C: cCRT → durvalumab (*n* = 318)	1 year	PFS (A vs. C)	16.7 vs. (not reported) vs. 15.6 A vs. C HR: 0.95 ^++^ CI: 0.77–1.19 B vs. C HR:0.84 CI: 0.69–1.04	A vs. C HR: 1.12 CI: 0.87–1.43 B vs. C HR: 0.97 CI: 0.76–1.24
CHORUS (NCT04905316)	II single arm	cCRT	cCRT + canakinumab → canakinumab + durvalumab (*n* = 32)	1 year	PFS	^7^	-
Keynote-799 (NCT03631784)	II 2 cohorts	cCRT	A (Sq or non-sq): 1C q3w chemoimmunotherapy → 2C q3w chemoimmunotherapy ^aa^ + TRT → pembrolizumab (*n* = 112) vs. B: non-sq: 1C q3w chemoimmunotherapy ^bb^ → 2 cycles of chemoimmunotherapy + TRT → pembrolizumab (*n* = 102)	1 year	ORR and incidence of grade ≥3 pneumonitis	A: 29.0 CI: 16.6–48.5 B: 45.3 CI: 17.9-NR)	A: 35.6 CI: 26.1–44.2 B: 56.7 CI: 41.1–NR
**Induction Immunotherapy Followed by cCRT**							
AFT 16 (NCT03102242)	II single arm	cCRT	4 cycles of induction atezolizumab → cCRT → atezolizumab (*n* = 62)	1 year	DCR	30.0 CI: 15.8–NE	NR ^8^

^1^ 12-month mOS 84.1%; ^2^ 12-month OS 84.1%;^3^ 18- and 24-month OS estimates: 82.1% and 76.6%, respectively; ^4^ 18- and 24-month OS estimates: 85.5% and 82.8%, respectively; ^5^ 1 yr PFS: 53.7% (95% CI 42.0–64.0%); ^6^ 2 yr OS rate 63.7% (95% CI 51.9–73.4%); ^7^ 11-month PFS: 75%; ^8^ OS rate at 24 months: 73.7% (95% CI 63.4–85.7%). * All arms received 1 year of consolidation therapy except where indicated. ^+^ 99% CI., ^++^ 96% CI. All CI are 95%, except where indicated; ^aa^ carboplatin + paclitaxel + pembrolizumab, ^bb^ cisplatin + pemetrexed +pembrolizumab. Abbreviations: PFS, progression-free survival; OS, overall survival; HR, Hazard ratio; CI, confidence interval; cCRT, concurrent chemoradiation therapy; CRT, chemoradiation therapy; sCRT, sequential chemoradiation therapy; HR, hazard ratio; mPFS, median progression-free survival; mOS, median overall survival; *n* = number of patients; NCT, National Clinical Trial; NR, not reached; NE, not estimable; OS, overall survival; vs., versus; y, years; TMDD, time to metastatic disease or death; BICR, blinded independent review; IA-ORR, investigator assessed objective response rates; sq, squamous; non-sq, non-squamous; TRT, thoracic radiation therapy; DCR, disease control rates.

**Table 2:** Trials of agents potentiating the activity of immune checkpoint inhibitors in the treatment of NSCLC.

National Clinical Trial Number	Immune Checkpoint Inhibitor	Other Agent(s)	Agent Type/Target
NCT06732401	Durvalumab	AZD6738	Ataxia telangiectasia and Rad3-related kinase
NCT06712316	BNT327	Standard chemotherapy	PD-L1 VEGF bispecific/inhibition of angiogenesis
NCT06700421	Adebrelimab	Apatinib	Inhibition of angiogenesis
NCT06667908	Durvalumab	Chemotherapy, radiation, JNJ-90301900	Functionalized hafnium oxide nanoparticles
NCT06617936	Tislelizumab	Recombinant human endostatin, chemotherapy	Inhibition of angiogenesis
NCT06512207	Sintilimab	Leuprorelin acetate	Anti-androgen
NCT06463665	Physician’s choice	Olvimulogene Nanivacirepvec and platinum-doublet	Oncolytic virus
NCT05967533	Unspecified/“standard of care”	Fermented wheat germ	Nutritional supplement
NCT05940532	Sugemalimab	Standard chemotherapy with ICI as induction followed by curative local therapy	N/A
NCT05798663	Atezolizumab	Tiragolumab	TIGIT
NCT05468242	Tislelizumab	Bevacizumab	Inhibition of Angiogenesis
NCT05334329	Atezolizumab	Genetically engineered NK cells	Genetically engineered NK cells
NCT04513925	Atezolizumab	Tiragolumab	TIGIT
NCT06623136	Toripalimab	ES102	OX40 agonist
NCT04198766	Pembroluzimab	INBRX-106	OX40 agonist
NCT05306847	Sintilimab	Anlotinib	Multi-targeted tyrosine kinase inhibitor
NCT05298423	Pembrolizumab	Vibostolimab	TIGIT
NCT05269381	Pembrolizumab	Neoantigen peptide-based vaccine	Personalized vaccine
NCT05248022	Sintilimab	Drug-eluting beads bronchial arterial chemoembolization	Drug-eluting beads bronchial arterial chemoembolization
NCT03257722	Pembrolizumab	Idelalisib	PI3K pathway
NCT05553834	Cemiplimab	Alirocumab	PCSK9 inhibition
NCT06385262	Cemiplimab	Alirocumab and chemotherapy	PCSK9 inhibition
NCT05198830	Durvalumab	TRC102, standard-of-care chemotherapy	Inhibition of base excision repair
NCT05096663	Pembrolizumab	N-803 (ALT-803)	IL-15 and receptor
NCT04940299	Ipilimumab and nivolumab	Tocilizumab	IL-6 receptor antagonist
NCT04699721	Nivolumab	Probiotics and standard chemotherapy	Microbiome
NCT04585490	Durvalumab	Tremelimumab and chemotherapy	CTLA-4 pathway
NCT04495153	“Standard of care”	CAN-2409 + prodrug	Oncogenic virus
NCT04287894	Durvalumab	Tremelimumab, chemoradiotherapy	CTLA-4 pathway
NCT04267237	Atezolizumab	RO7198457	Personalized vaccine
NCT04007744	Pembrolizumab	Sonidegib	Hedgehog signaling pathway
NCT03801902	Durvalumab	Molalizumab (IPH2201)	NGK2A (alternative immune checkpoint receptor)
		Olecumab (MEDI9447)	Adenosine
NCT03520686	“Standard of care”	N-803	IL-15 superagonist complex
NCT03048500	Nivolumab	Metformin	AMPK pathway
NCT02983578	Durvalumab	Danvatirsen	STAT3
NCT05177497	SHR-1701		Bifunctional fusion protein composed of a mAb against PD-L1 fused with the extracellular domain of TGF-β receptor II
NCT02403193	Spartalizumab	PBF-509	Adenosine A2a receptor antagonist
NCT05211895	Durvalumab	Domvanalimab	TIGIT
NCT04380636	Pembrolizumab	Olaparib	PARP
NCT06540950	Unspecified	Vinorelbine	Anti-microtubular agent
NCT05687266	Durvalumab	Datopotamab Deruxtecan, carboplatin	TROP2
NCT01454102	Nivolumab	Gemcitabine and cisplatin	Multiple pre-existing agents
		Pemetrexed and cisplatin	
		Bevacizumab	
		Erlotinib	
		Ipilimumab	

Abbreviations: AMPK, AMP-activated protein kinase; CTLA-4, cytotoxic T-lymphocyte-associated protein 4; ICI, immune-checkpoint inhibitor; IL, interleukin; N/A, not applicable; NCT, National Clinical Trials; NK, natural killer; PARP, poly ADP ribose polymerase; PCSK9, Proprotein Convertase Subtilisin/Kexin Type 9; PI3K, phosphatidylinositol-3-kinase; STAT3, signal transducer and activator of transcription 3; TIGIT, T cell immunoreceptor with Ig and ITIM domains; TROP2, trophoblast cell-surface antigen 2.
